# Resolutions on the Death of Dr. James W. White

**Published:** 1891-08

**Authors:** B. C. Nash

**Affiliations:** Secretary


### Obituary.

### RESOLUTIONS ON THE DEATH OF DR. JAMES W. WHITE.

At a meeting of the First District Dental Society of the State of New York, held June 9, 1891, the following resolutions were unanimously adopted:

Whereas, The First District Dental Society of the State of New York has learned, with feelings of deep regret, of the death of Dr. James W. White, of Philadelphia, an eminent and learned member of the dental profession,

*Resolved*, That in the death of Dr. White we recognize the fact that we have lost one who, by his labors in dental journalism and literature, has contributed largely to the advancement and elevation of dentistry as a profession.

*Resolved*, That as an evidence of respect to the late Dr. James W. White, and of appreciation of his valuable services to our profession, these resolutions be entered upon the records of the Society, a copy be sent to his family and to the dental journals for publication.

B. C. Nash,
*Secretary.*

